# Development of a wound management pathway at a small Rural Health Service

**DOI:** 10.1186/1757-1146-8-S2-P14

**Published:** 2015-09-22

**Authors:** Louine Robinson, Lisa Pryor, Linda Earl

**Affiliations:** 1 Beechworth Health Service, Beechworth, VIC, 3747, Australia

**Keywords:** Wound management, industry partnership, small rural Health Service

## Background

Beechworth Health Service (BHS) formed a Wound Management Working Party (WMWP) in 2011 to improve wound management and client outcomes. Existing expertise and systems were utiliized in conjunction with public and private agencies to ensure best practice and continuity of care for acute, aged care and community clients.

## Process

The WMWP consisted of executive, allied health, unit managers, accounts and clinical staff to ensure the committee had organisation wide buy-in and could act on recommendations made. In 2012, BHS partnered with an industry consultative Wounds Improvement Program to review current systems and conduct a wound prevalence audit of acute, residential and community clients. A report of recommendations was produced which included: standardisation of procedures; policies; wound products; documentation; an education program for all staff involved in wound care to support change and embed new practice. The report recommendations were implemented early 2013. Additional education and resources were accessed from the Victorian Department of Health initiative, Connected Wound Care program. As the wound management pathway became formalised at BHS, it was recognised the working party's role was finished and the Wound Management Committee (WMC) took over monitoring and reviewing the wound management pathway. The organisation wide audit is now conducted annually. There have been 3 to date.

## Findings

The partnership with industry has resulted in improved client and organisational outcomes. The results of the 2014 wound prevalence audit found that the incidence and severity of wounds had decreased since 2012.

## Conclusions

The establishment of a partnership with BHS and industry through the Wound Improvement Program has been shown to improve wound management outcomes. Small Rural Health Services can utilise resources from public and private sectors that can facilitate the delivery of best practice wound care. The use of common resources has: facilitated a regionally consistent message and care to clients; created networks between health services in the Hume region formalised in the Wounds in Hume Improvement Committee.

